# A comparative in vitro study on monomer release from flash-free or conventional bonding systems

**DOI:** 10.1007/s00784-025-06250-3

**Published:** 2025-03-06

**Authors:** Baris Can Telatar, Gul Yildiz Telatar

**Affiliations:** 1 Department of Orthodontics, Faculty of Dentistry, Akdeniz University, Antalya, Turkey; 2 Department of Restorative Dentistry, Faculty of Dentistry, Burdur Mehmet Akif Ersoy University, Burdur, Turkey

**Keywords:** APC flash-free, Monomer release, Orthodontics, Bonding

## Abstract

**Objectives:**

To examine the amount of residual monomer released from adhesive-precoated flash-free brackets and compare it to traditional light cure and chemical cure adhesives.

**Materials and methods:**

Sixty stainless steel brackets were bonded on upper premolar teeth using three groups of adhesive systems: Adhesive-precoated flash-free system, Transbond XT light cure, Sia chemical cure. The extracts of 0.5 ml of liquid samples were taken on the first, 7th, 21st, and 35th days. To determine the amount of residual monomer release, extracted samples were analyzed using a liquid chromatography device.

**Results:**

Urethane-dimethacrylate, triethylene glycol dimethacrylate, hydroxyethyl methacrylate and Bisphenol A-glycidyl methacrylate monomers were released from all study groups. The amount of total monomer release except hydroxyethyl methacrylate was detected as greater in adhesive-precoated flash-free system. Among the study groups, the chemical cure group showed the highest hydroxyethyl methacrylate monomer release.

**Conclusions:**

This in vitro study indicate that the APC flashless system exhibited the greatest release of residual monomers, suggesting that its possible cytotoxic effects need to be evaluated prior to clinical application.

**Clinical relevance:**

This is the first study that evaluates the residual monomer release from adhesive-precoated flash-free brackets. The novel bracket system has a different mesh structure at the bracket base compared to traditional systems with different chemical components which might alter the amount of residual monomer release. To investigate the harmful effects of adhesives used in orthodontic applications is very important for the health of the patient and the orthodontist.

**Supplementary Information:**

The online version contains supplementary material available at 10.1007/s00784-025-06250-3.

## Introduction

The fixed orthodontic treatment is a time-consuming process, and an average fixed treatment ends in 24.9 months approximately [1]. It is well known that the oral environment alters the mechanical structure of the dental materials [2]. The thermal and pH changes, oral microbiota, enzymatic reactions, and generated forces during chewing are suspected factors in structural deterioration and biodegradation of the materials [3]. 

Polymers are widely used in orthodontic practice, and polymers consist of many kinds of appliances, such as aesthetic brackets, elastomeric ligatures, chains, and orthodontic composite resin materials [4]. Among these polymeric materials, orthodontic composites are considered in terms of residual monomer release particularly [5, 6]. 

Composite resin materials contain three main components. These are the organic phase which includes monomers, the inorganic phase which determines the fillers, and the coupling agent between these two phases [7]. The characteristics of the composites, such as stiffness and polymerization shrinkage, mainly differ depending on the composition and contents of these three main components.

Cross-linked monomers make the polymers mechanically more robust [5]. Therefore, urethane dimethacrylate (UDMA), triethylene glycol dimethacrylate (TEGDMA), hydroxyethyl methacrylate (HEMA) and Bisphenol A-glycidyl dimethacrylate (BisGMA) are cross-linked monomers that are used in dental composites [5]. BisGMA and UDMA are the most used base monomers. BisGMA and UDMA are high-molecular-weight monomers that form a high viscosity, are more resistant to biodegradation, and have high stiffness and low wettability [8]. Therefore, it has been used in combination with low-molecular-weight comonomers, such as TEGDMA and HEMA, to facilitate clinical application [9, 10]. 

Orthodontic adhesives contain both low and high molecular weight monomers. Polydorou et al., [11] reported that while 50% of the total amount of low molecular weight monomer that was eluted after polymerization was released during the first 24 h of storage, the elution of high molecular weight monomer continued to increase compared to the low molecular monomers even after 4 weeks of storage.

In 2013 adhesive-precoated (APC) flash-free system was introduced with advantages of easier and faster bonding procedure compared to conventional systems due to eliminating the need of excessive adhesive removal [12]. The nonwoven polypropylen mesh soaked by a low- viscosity resin at the bracket base provide smooth, more regular transition from adhesive to enamel surface [13]. 

In the present study, we tested three different bonding systems used in orthodontic practice. These are the chemical cure, light cure, and APC flash-free system, which is newly introduced. There are numerous studies conducted on chemical and light cure composites in terms of residual monomer release [5, 6, 14]. Gioka et al., [6] evaluated the monomer release and reported no difference between chemical and light cure groups while Hezenci et al., [5] and Eliades et al., [14] demonstrated greater monomer release in chemical cure group. The APC flash-free system was studied with regard to bracket bonding failure rates, adhesive remnant index, enamel demineralization effects, and bonding time [13]. However, there is no study that assesses the residual monomer release for APC flash-free system which has a nonwoven mesh at the bracket base.

Hence, the aim of the present in vitro study is to investigate the release of UDMA, TEGDMA, HEMA and BisGMA from three different bonding systems using high-performance liquid chromatography (HPLC) after polymerization. The hypothesis of the study is chemical cure, light cure, and APC flash-free system cause differences in the amount of residual monomer release.

### Materials and methods

G*Power package software was used to estimate the number of samples to be used in the study. It was determined that at least 12 samples were required for the each test groups, with α = 0.05 type I, and β = 0.05 type II error rates for 95% power in f = 0.80 effect size. To cover possible data loss, a decision was made to utilize 20 samples for each group.

Sixty extracted upper premolar teeth and sixty upper premolar brackets, 40 were 3 M Victory series low profile MBT brackets (3 M Unitek, Monrovia, CA), 20 were 3 M Victory series low profile APC flash free brackets (3 M Unitek, Monrovia, CA) were used in this study. The root for each tooth was resected and after removing the root system, soft tissue remnants were eliminated from the teeth surfaces, and the teeth were polished with nonfloridated pumice. After the polishing procedure, all teeth were cleaned up under running tap water to remove pumice residues. The teeth were immersed in a 5% sodium hypochlorite during first 24 h for disinfection purposes and stored in distilled water until use. Three commercially available and routinelly used adhesive materials were included in the study. Although not mentioned in the safety data sheets UDMA and HEMA release were also tested in case the manufacturers prepared the safety data sheets incompletely. Before the bonding procedure, the teeth were randomly assigned into three groups according to the adhesive system used. The adhesive systems used in the study are shown in Table 1.

**Table 1:** The adhesive systems used in the study

Product	Manufacturer	Material Safety Data Sheet
Transbond XT Primer	3 M, St. Paul, MN, USA	Bisphenol A Diglycidyl Ether Dimethacrylate (BISGMA), Triethylene Glycol Dimethacrylate (TEGDMA), 4-(Dimethylamino)-Benzeneethanol
Transbond XT Light Cure Paste	3 M, St. Paul, MN, USA	Silane Treated Quartz, Bisphenol A Diglycidyl Ether Dimethacrylate (BISGMA), Bisphenol A Dimethacrylate, Silane Treated Silica, Diphenyliodonium Hexafluorophosphate, Triphenylantimony
Sia Chemical Cure Primer	Sia Orthodontic Manufacturer, ITALY	Bisphenol A Diglycidyl Methacrylate, Triethylene Glycol Dimethacrylate
Sia Chemical Cure Paste	Sia Orthodontic Manufacturer, ITALY	Bisphenol A Diglycidyl Methacrylate, Triethylene Glycol Dimethacrylate
APC Flash Free	3 M, St. Paul, MN, USA	Bisphenol A Bis (2 Hydroxyethyl Ether) Dimethacrylate, Bisphenol A Diglycidyl Ether Dimethacrylate (BisGMA)

The bonding procedures were performed according to the manufacturer’s instructions. Sia group had self curing resin, while APC flash free group and Transbond XT light cure group were polimerized using Valo Cordless LED device (Ultradent - South Jordan, United States) (3200 mW/cm2 × 6 s). The tip of the light curing device gently and perpendicularly touched the bracket surface ensuring that its position was not altered. After the bonding procedure, all specimens were kept at 37,8 C and 50% humidity for five minutes for adequate polymerization as suggested [5]. All samples were immersed in sterile glass tubes containing 5 ml of ethanol solution (99,99% /v HPLC grade) immedietally after bonding procedure to achieve accelerated aging. The ethanol was preferred due to its degradation and aging effects [15]. Each specimen in the glass tubes was shaken once a day during the analysis period. To determine the amount of residual monomer release, a HPLC device (Agilent Technologies, California, USA) was used. The HPLC device consisted of the following components: A gradient pump (Agilent 1260 series, California, USA), an injector with a 20 µl sample loop (Agilent 1260 series, California, USA), a reversed-phase C18 analytical column (250 × 4.6 mm 5 μm) (Usem, Kayseri, TURKEY), and an absorbance detector (Agilent 1260 series, California, USA). The assessments were performed under the following conditions: acetonitrile/water (65:35 v/v) mobile phase; isocratic elution mode; 1 ml/min flow rate; and detection at 210 nm. The extracts of 0.5 ml of ethanol solution were taken from the glass tubes on the first, 7th, 21st, and 35th days and filtered via 0.22 μm nylon filter paper, and the filtered solution was injected into opaque glass vials.

The known concentrations of UDMA, TEGDMA, HEMA and BisGMA were used for the calibration of the column. For assessing the released monomer in ethanol solution, the linear fittings of the calibration curves were used. The HPLC evaluations were duplicated each time, and an average value was obtained.

### Statistical analysis

Statistical evaluation was performed with the IBM SPSS 25.0 (SPSS Inc., Chicago, IL, America) program. The normal distribution test was evaluated with the Kolmogorov-Smirnov Test. A two-way repeated measures ANOVA and posthoc Tukey test statistics were used to determine whether the amount of released residual monomer differed in terms of adhesive systems used and 1, 7, 21, and 35th time periods. *P* <.05 was considered sufficient for statistical significance.

## Results

The accumulated levels of each residual monomer at each time point are presented in tables. The amounts of each monomer release were elaborated separately to show behavioral characteristics.

### UDMA

**Fig. 1:**
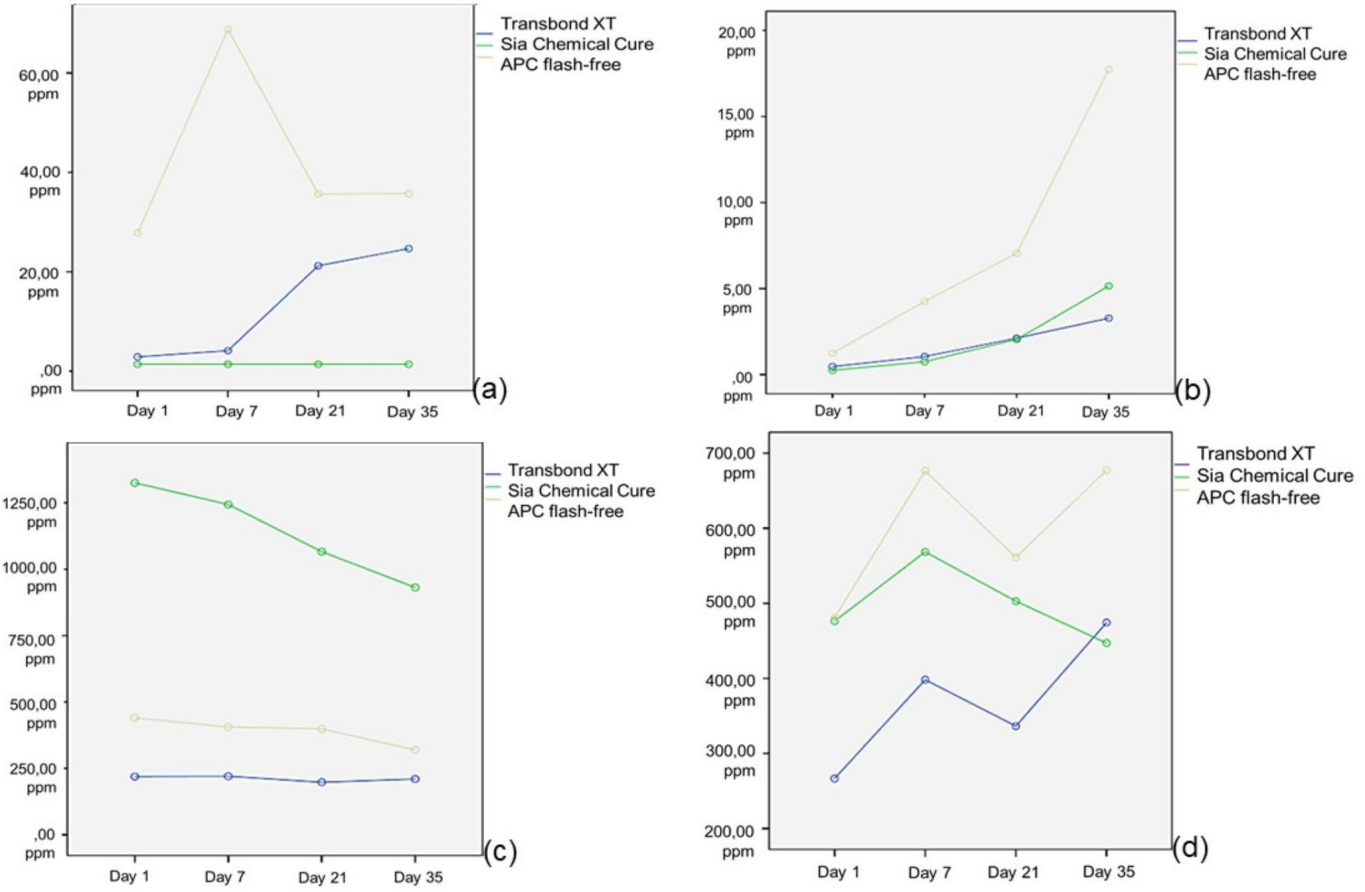
Q1

The amount of UDMA release for each time period is presented in Fig. 1a. The greatest UDMA release was detected in the APC flash-free group and the lowest in the chemical cure group. The APC flash-free group showed statistically higher UDMA release than the other two groups on all evaluated days (*p* <.01). UDMA release in the chemical group was the same and minimal at all time points. While the greatest UDMA release in the Transbond XT group was on days 21 and 35, the greatest UDMA release in the APC flash-free group was on day 7 (*p* <.01). The limits of detection (LOD) and limits of quantification (LOQ) were 0.041 ppm and 0.123 ppm, respectively. (Table 2)

**Table 2:** Means and standard deviations (in ppm) of the UDMA readings in each group evaluated on days 1, 7, 21, and 35

Adhesive	Day 1, Mean (SD)	Day 7, Mean (SD)	Day 21, Mean (SD)	Day 35, Mean (SD)
Transbond XT	2.88(0.00)^A,1^	4.140.00)^A,1^	21.21(3.76)^B,1^	24.66(5.84)^C,1^
Sia Chemical Cure	1.40(0.00)^A,1^	1.40(0.00)^A,1^	1.40(0.00)^A,2^	1.40(0.00)^A,2^
APC Flash Free	27.83(11.70)^A,2^	68.73(20.49)^B,2^	35.64(12.69)^A, C,3^	35.71(11.40)^C,3^
LOD: 0.041			LOQ: 0.123	

SD indicates standard deviation; UDMA, urethane dimethacrylate; LOD, limits of detection; LOQ, limits of quantification.Note: Same superscript capital letters indicate an insignificant difference (*P* =.05) within the same row; same superscript numbers indicate an insignificant difference (*P* =.05) within the same column

### TEGDMA

The amount of TEGDMA release for each time period is presented in Fig. 1b. The greatest TEGDMA release was in the APC flash-free group at all time points (*p* <.01). TEGDMA release increased over time for each group from the beginning. (*p* <.01). While the Tansbond XT and chemical groups exhibited similar TEGDMA values ​​on the 35th day, the greatest release was detected in the APC flash-free group compared to the other two groups. (*p* <.01). The LOD and LOQ were calculated as 1.63 ppm, and 4.9 ppm, respectively for TEGDMA. (Table 3)

**Table 3:** Means and standard deviations (in ppm) of TEGDMA readings in each group evaluated on days 1, 7, 21, and 35

Adhesive	Day 1, Mean (SD)	Day 7, Mean (SD)	Day 21, Mean (SD)	Day 35, Mean (SD)
Transbond XT	0.47(0.00)^A,1^	1.05(0.00)^B,1^	2.12(0.00)^C,1^	3.28(0.00)^D,1^
Sia Chemical Cure	0.25(0.00)^A,2^	0.75(0.00)^B,2^	2.05(0.00)^C,2^	5.15(0.00)^D,1^
APC Flash Free	1.25(0.00)^A,3^	4.25(0.00)^B,3^	7.05(0.00)^C,3^	17.73 (12.28)^D,2^
LOD:1.63			LOQ: 4.9	

SD indicates standard deviation; TEGDMA, triethylene glycol dimethacrylate; LOD, limits of detection; LOQ, limits of quantification.Note: Same superscript capital letters indicate an insignificant difference (*P* =.05) within the same row; same superscript numbers indicate an insignificant difference (*P* =.05) within the same column

### HEMA

The amount of HEMA release for each time period is presented in Fig. 1c. It was observed that there was a similar release in all time periods according to the initial measurements in both the Transbond XT and APC flash-free groups. There was no difference between the APC flash-free and Transbond XT groups. However, the highest HEMA release was detected in the chemical cure group (*p* <.01). The LOD and LOQ were 0.069 ppm and 0.207 ppm, respectively. (Table 4)

**Table 4:** Means and standard deviations (in ppm) of the HEMA readings in each group evaluated on days 1, 7, 21, and 35

Adhesive	Day 1, Mean (SD)	Day 7, Mean (SD)	Day 21, Mean (SD)	Day 35, Mean (SD)
Transbond XT	219,11(199,39)^A,1^	220.40(190.09)^A,1^	198.04(166.54)^A,1^	210.03(180.90)^A,1^
Sia Chemical Cure	1325,09 (515.11)^A,2^	1243.66(477.21)^B,2^	1066.10(389.28)^C,2^	931.29(338.14)^D,2^
APC Flash Free	440,46 (694.10)^A,1^	405.79(644.12)^A,1^	398.76(583.21)^A,1^	319.63(447.04)^A,1^
LOD:0.069			LOQ:0.207	

SD indicates standard deviation; HEMA, hydroxyethyl methacrylate; LOD, limits of detection; LOQ, limits of quantification.Note: Same superscript capital letters indicate an insignificant difference (*P* =.05) within the same row; same superscript numbers indicate an insignificant difference (*P* =.05) within the same column

### BisGMA

The amount of BisGMA release for each time period is presented in Fig. 1d. In the initial measurements, all adhesive systems showed similar monomer release. On the 35th day of the evaluation, statistically more residual monomer release was observed in the APC flash-free adhesive system compared to the other groups. (*p* <.01). On the 7th and 21st days of the measurement, the BisGMA release of the APC flash-free group was similar to the chemical cure adhesive group, and more residual monomer release was achieved compared to the Transbond XT adhesive system. The LOD and LOQ were calculated as 0.051 ppm, and 0.154 ppm, respectively for BisGMA. (Table 5)

**Table 5:** Means and standard deviations (in ppm) of the BisGMA readings in each group evaluated on days 1, 7, 21, and 35

Adhesive	Day 1, Mean (SD)	Day 7, Mean (SD)	Day 21, Mean (SD)	Day 35, Mean (SD)
Transbond XT	266.60(157.81)^A,1^	398.18(142.46)^B,1^	336,45(146.93)^C,1^	474,35(160.09)^D,1^
Sia Chemical Cure	476.38(107.27)^A,1^	568.50(157.53)^B,1,2^	502,83(131.87)^A, B,1,2^	447,08(137.05)^A,1^
APC Flash Free	480.89(578.79)^A,1^	676.42(502.87)^B,2^	561,40(449.40)^A,2^	677,27(354.62)^B, C,2^
LOD: 0.051			LOQ:0.154	

BisGMA indicates bisphenol A-glycidyl dimethacrylate; SD, standard deviation; LOD, limits of detection; LOQ, limits of quantification.Note: Same superscript capital letters indicate an insignificant difference (*P* =.05) within the same row; same superscript numbers indicate aninsignificant difference (*P* =.05) within the same column

## Discussion

Several studies revealed residual monomer release after the polymerization of polymeric materials used in orthodontic practice [3, 5, 6, 14]. The amount of the released residual monomer differs depending on the used light curing device, the content of the cured material, exposure time, distance from the material to the light curing device, temperature of the ambiance, and oxygen in the environment [3, 5]. 

Alshali et al. reported that the highest monomer release occurred right after the resin material was cured and that this gradually decreased over time [16]. Moreira et al. and Sideridou et al. reported residual monomer release until 30th days after polymerization [17, 18]. Similar to Hezenci et al. [5], the residual monomer examinations were performed on the first, 7th, 21st, and 35th days.

The oral environment has potential degradation effects on dental composites caused by salivary [19]. The degradation effect of saliva is imitated with various solvents, such as water, artificial saliva, acetonitrile, ethanol, and methanol for in vitro studies [20]. It was reported that ethanol is the most commonly used solvent in the degradation of various adhesives used in dentistry [21]. Moreover, it was reported that ethanol is a more aggressive solvent and composite elutes more monomer in organic immersion media compared to water or water based media [22]. The importance of the choice of true extraction media was emphasized due to protein binding which leads to false negative results [23]. In this study, 99.9% ethanol recommended by the US Food and Drug Administration (FDA) was used to reflect oral conditions as realistically as possible [5]. 

Type of the light curing device and curing time can contribute the elution of residual monomer release. Carvalho et al., [24] and Ak et al., [25] reported less residual monomer release with LED devices compared to halogen devices. Hence, we used LED device in the the present study. It has been reported that the amount of residual monomer release decreases as the irradiation time increases [26]. Besides this, reducing the curing time was considered safer regarding the temperature changes in the pulp chamber [27]. 

An accurate definition of the analyte is crucial for creating a dependable study framework aimed at identifying monomers and the estimation of the limit of detection (LOD), which should be as low as technically achievable [28]. Identification of the released components is usually carried out by using HPLC, liquid chromatography-mass spectrometry (LC-MS), and gas chromatography (GC) [29]. It is suggested that HPLC and LC-MS are more suitable methods for samples with high molecular weight such as Bis-GMA and UDMA and/or with high potential to deteriorate when it is heated. Additionally, the HPLC is efficient and accurate in the detection of monomer elution from the sample [30, 31]. Since HPLC have been used for monomer release in the recent studies of orthodontics, this method was also preferred in our study [3, 5]. 

Although residual monomer release has been widely assessed for orthodontic adhesives [4, 5, 14], this is the first study examining the amount of residual monomer released from the novel bracket system APC flash-free. The material safety data sheets are not exhaustive and manufacturers do not declare all components of the dental materials [3, 32]. Therefore, various substances not stated in the safety data sheets such as UDMA and HEMA were detected in all study groups. Hezenci et al. noted a greater amount of UDMA, TEGDMA, HEMA and BisGMA in the chemical cure adhesive group than in the light cure group [5]. Eliades et al., [14] reported a significantly lower residual monomer release in light-cured adhesives than in chemical curing systems. Similar to Eliades et al., Jagdish et al. [33] reported more residual monomer release for the chemical cure group than the light cure group. In the present study, although the Transbond XT group showed lower residual monomer release than the chemical cure group, we found the highest residual monomer release occurred in the novel light cure bracket system APC flash-free.

Adhesive systems containing high filler volume and high molecular weight monomer content may provide thicker layers as a result of higher viscosity which cause decrease in conversion rates [34]. Hence, a firm finger pressure was applied on the brackets for achieving consistent thickness. The APC flash free system has a polypropylene mesh at the bracket base with different stuctural and chemical components which we attribute the higher monomer release. Besides this, greater monomer elution was observed in chemical cure group compared to light cure group may be explained by the increased extent of the oxygene inhibition layer during prolonged curing reaction.

The cytotoxic effects of each monomer were ranked from high to low as BisGMA, UDMA, TEGDMA, and HEMA [35]. The determined cytotoxic doses of BisGMA, UDMA, TEGDMA, and HEMA were 4.78 ppm, 8.17 ppm, 37.75 ppm, and 520 ppm, respectively [36–38]. According to the threshold values; BisGMA, UDMA, and HEMA reached the levels that would have harmful effects. UDMA showed higher than threshold values for Transbond XT, and APC flash-free groups whereas TEGDMA did not reach the effective dose concentration in any study group. HEMA showed the highest release in the chemical cure group that is outside of safe limits whereas BisGMA reached the cytotoxic levels for each group. APC flash-free group showed the most amount of release for UDMA, TEGDMA and BisGMA.

It was revealed that low molecular weight monomers, such as HEMA and TEGDMA, have a high speed release rate compared to high molecular weight monomers, BisGMA and UDMA [5]. In the present study, it was observed that UDMA showed a slower release rate for the Transbond XT group, and HEMA showed a faster release rate for all study groups which is consistent with the literature.

Since the degradation products of the polymers, all plastic packaging as food contact material has been restricted in the EU [39]. BisGMA is a BPA based monomer and BPA was banned in plastic for baby bottles. The 2023 European Food Safety Authority reported the tolerable daily intake of BPA as 0.2ng/kg body weight per day [40]. Many studies reported negative biological effects of residual monomers [6, 35–38, 41]. It was reported that the estrogenicity effect of BPA adversely affects the growth of the children and homeostasis of adults [41]. Besides this, it was reported that various polymeric derivates have a potential risk for allergic, mutagenic, and carcinogenic effects on tissue and cell levels [6]. In this respect evaluating the potentially harmful effects of the daily used orthodontic materials is very essential for preventing the developmental and reproductive toxicity [4]. 

This study has several limitations. The study was conducted under in vitro conditions and the washing effect of the saliva could not be evaluated. In the present study, ethanol was used, which tends to release more residual monomer. One more limitation was the use of an HPLC instrument for testing purposes. The use of LC-MS, known for its superior sensitivity and specificity as well as its quicker processing time, might have been a more suitable choice. The follow-up time was restricted to 35 days. Therefore, an in vivo study with a longer follow-up time is recommended.

## Conclusion

Although all materials released the residual monomer above safety thresholds, the most residual monomer release was shown for the APC flash-free system, and orthodontists should be aware of the potential cytotoxic effects before using them in clinical practice. Since Transbond XT released the least residual monomer, it could be considered the safest choice in the bonding procedure. Ethanol is an aggressive solution for monomer elution hence it is recommended to conduct a clinical prospective study.

## Electronic supplementary material

Below is the link to the electronic supplementary material.

Supplementary Material 1

Supplementary Material 2

## Data Availability

No datasets were generated or analysed during the current study.
